# Association of artificially sweetened and sugar-sweetened soft drinks with β-cell function, insulin sensitivity, and type 2 diabetes: the Maastricht Study

**DOI:** 10.1007/s00394-019-02026-0

**Published:** 2019-09-05

**Authors:** Louise J. C. J. den Biggelaar, Simone J. S. Sep, Andrea Mari, Ele Ferrannini, Martien C. J. M. van Dongen, Nicole E. G. Wijckmans, Miranda T. Schram, Carla J. van der Kallen, Nicolaas Schaper, Ronald M. A. Henry, Marleen M. van Greevenbroek, Coen D. A. Stehouwer, Simone J. P. M. Eussen

**Affiliations:** 1 Department of Epidemiology, Maastricht University, P.O. Box 616, 6200 MD Maastricht, The Netherlands; 2 Cardiovascular Research Institute Maastricht (CARIM), Maastricht University, Maastricht, The Netherlands; 3 Department of Rehabilitation Medicine, Maastricht University, Maastricht, The Netherlands; 4 Care and Public Health Research Institute (CAPHRI), Maastricht University, Maastricht, The Netherlands; 5 C N R Institute of Neuroscience, Padua, Italy; 6 C N R Institute of Clinical Physiology, Pisa, Italy; 7 Heart and Vascular Centre, Maastricht University Medical Centre+, Maastricht, The Netherlands

**Keywords:** Artificially sweetened beverages, Sugar-sweetened beverages, Soft drink, Juice, Beta-cell function, Insulin sensitivity, T2D

## Abstract

**Purpose:**

Artificially sweetened and sugar-sweetened beverage consumptions have both been reported to be associated with type 2 diabetes mellitus (T2D) risk. The aim of the current study was to investigate the potential underlying associations with dynamic pancreatic β-cell function (BCF) and insulin sensitivity.

**Methods:**

We evaluated cross-sectional associations in 2240 individuals (mean ± SD age 59.6 ± 8.18, 49.4% male, 21.9% T2D) participating in a diabetes-enriched population-based cohort. Artificially sweetened and sugar-sweetened soft drinks and juice consumption were assessed by a food-frequency questionnaire. Glucose metabolism status, insulin sensitivity, and BCF were measured by a seven-point oral glucose tolerance test. Regression analyses were performed to assess associations of artificially and sugar-sweetened beverage consumption with measures of glucose homeostasis. Associations were adjusted for potential confounders, and additionally with and without total energy intake and BMI, as these variables could be mediators.

**Results:**

Moderate consumption of artificially sweetened soft drink was associated with lower β-cell glucose sensitivity [standardized beta (95% CI), − 0.06 (− 0.11, − 0.02)], total insulin secretion [*β* − 0.06 (− 0.10, − 0.02)], and with lower β-cell rate sensitivity [odds ratio (95% CI), 1.29 (1.03, 1.62)] compared to abstainers. Daily artificially sweetened soft drink consumption was associated with lower β-cell glucose sensitivity [*β* − 0.05 (− 0.09, 0.00)], and total insulin secretion [*β* − 0.05 − 0.09, − 0.01)] compared to abstainers.

**Conclusions:**

Moderate and daily consumption of artificially sweetened soft drinks was associated with lower BCF, but not with insulin sensitivity. No evidence was found for associations of sugar-sweetened soft drink and juice consumption with BCF or insulin sensitivity in this middle-aged population. Prospective studies are warranted to further investigate the associations of artificially and sugar-sweetened beverage consumption with non-fasting insulin sensitivity and multiple BCF aspects.

**Electronic supplementary material:**

The online version of this article (10.1007/s00394-019-02026-0) contains supplementary material, which is available to authorized users.

## Introduction

The epidemic rise in the prevalence of type 2 diabetes mellitus (T2D) is partly caused by unhealthy dietary habits, including the consumption of free sugars [1]. A major source of free sugars is sugar-sweetened beverages [1, 2]. A recent meta-analysis [3] showed that sugar-sweetened beverage consumption was associated with a higher risk of T2D (RR 1.21, 95% confidence interval 1.12–1.31, *n* = 10 studies) per 250 g/day increase of sugar-sweetened beverage. Furthermore, sugar-sweetened beverage consumption has been positively associated with prediabetes [4] and inversely with insulin sensitivity in observational studies [4–7].

Artificially sweetened beverages have been marketed successfully as a healthy alternative for sugar-sweetened beverage consumption, aimed at controlling body weight and glucose homeostasis [8]. However, results of the previously mentioned meta-analysis [9] revealed that artificially sweetened beverage consumption is associated with an increased risk of T2D with a relative risk of 1.25 (95% confidence interval 1.18–1.33) per serving/day without, and 1.08 (1.02–1.15) after adjustment for adiposity, based on data of ten prospective cohorts [9].

The results of prospective studies varied from nonsignificant to positive associations of artificially sweetened beverage consumption with prediabetes [6, 10] and insulin resistance [5, 6, 10]. Only one study focused on the association of artificially sweetened beverage consumption with BCF (HOMA-β) and showed an inverse association with BCF in lean individuals only [6].

Despite the fact that BCF and insulin sensitivity provide important insights into the ethology of T2D, only a few studies [4–6] have focussed on the association of artificially and sugar-sweetened beverage consumption with BCF and insulin sensitivity. Furthermore, these few studies mainly investigated BCF or insulin sensitivity based on fasting conditions, while oral glucose tolerance test (OGTT)-based measures physiologically reflect the insulin secretory response of pancreatic beta cells to oral glucose ingestion [11–13]. Therefore, the aim of the current cross-sectional study (*n* = 2240) was to investigate the associations of artificially sweetened and sugar-sweetened soft drinks, juice, and total sugar-sweetened beverage consumption with OGTT-based measures of BCF and insulin sensitivity as primary outcomes and with prediabetes and T2D as secondary outcomes.

## Methods

### The Maastricht Study design and population

We used data from the Maastricht Study, an observational prospective population-based cohort study. The rationale and methodology have been described previously [14]. In brief, the study focuses on the etiology, pathophysiology, complications, and comorbidities of T2D and is characterized by an extensive phenotyping approach. Eligible for participation was all individuals aged between 40 and 75 years and living in the southern part of The Netherlands. Participants were recruited through mass media campaigns and from the municipal registries and the regional Diabetes Patient Registry via mailings. Recruitment was stratified according to known T2D status, with an oversampling of individuals with T2D, for reasons of efficiency. The study has been approved by the institutional medical ethical committee (NL31329.068.10) and the Minister of Health, Welfare and Sports of The Netherlands (Permit 131088-105234-PG). All participants gave written informed consent.

The present report includes cross-sectional data from the first 3451 participants, who completed the baseline survey between November 2010 and September 2013. The examinations of each participant were performed within a time window of 3 months [14]. For the present analyses, we excluded individuals who had another type diabetes than type 2 (*n* = 41), used insulin (*n* = 220), suffered from cancer (*n* = 189), had not filled out a food-frequency questionnaire (FFQ) (*n* = 185) or had reported implausible total energy intake (< 800 or > 4200 kcal/day for men and < 500 or > 3500 kcal/day for women, *n* = 56) [15]. Finally, complete case analyses were performed; therefore, participants with a missing fasting or 120 min post glucose load blood sample, overall less than 5 OGTT blood sampling points (*n* = 250), or with missing values in covariates (*n* = 326) were excluded. This resulted in a study population of 2240 individuals for the outcome measures BCF and insulin sensitivity. For the analyses with prediabetes as an outcome, individuals with T2D were additionally excluded (*n* = 475). For the analyses with newly diagnosed T2D as an outcome, we excluded individuals who were previously diagnosed with T2D (*n* = 384). This resulted in a study population of 1765 and 1856 individuals for the outcome measures prediabetes and newly diagnosed T2D, respectively.

### Data collection

#### Glucose metabolism

To determine glucose metabolism status, all participants, except those who used insulin, underwent a standardized 2-h 75 g oral glucose tolerance test (OGTT) after an overnight fast, venous blood samples were collected before, and 15, 30, 45, 60, 90 and 120 min after oral glucose load.

Glucose metabolism status was defined according to the WHO 2006 criteria (9): NGM (fasting plasma glucose < 6.1 mmol/l and 2 h post-load plasma glucose < 7.8 mmol/l), prediabetes (fasting plasma glucose levels of 6.1–6.9 mmol/l and/or 2 h post-load glucose levels of 7.8–11.1 mmol/l) and T2DM (fasting plasma glucose ≥ 7.0 mmol/l and/or 2 h post-load glucose ≥ 11.1 mmol/l) [16]. In the present study, individuals with impaired fasting glucose and/or impaired glucose tolerance were combined into one category—that is, prediabetes. In case individuals were on diabetes medication but did not have type 1 diabetes they were classified as having T2D [14].

Plasma for the assessment of insulin and C-peptide levels was collected in EDTA tubes, stored on ice, separated after centrifugation (3000×*g* for 15 min at 4 °C), and stored at − 80 °C until the assays were performed. The time between collection and storage was < 2 h. Insulin and C-peptide were measured in never-thawed plasma by use of a custom duplex array of MesoScale Discovery (MesoScale Discovery, Gaithersburg, MD, USA, http://www.mesoscale.com). In short, 96 well-plates, with capture antibodies against insulin and C-peptide patterned on distinct spots in the same well, were supplied by the manufacturer. Samples (10 µl/well), detection antibodies and read buffer for electrochemiluminescence were applied according to manufacturer’s instruction and plates were read using a SECTOR^®^ 2400 Imager. Detection ranges of the assay were 35–25,000 pg/ml for insulin and 70–50,000 pg/ml for C-peptide. Interassay coefficients of variation for insulin and C-peptide were 10.1% and 8.2%, respectively. Insulin and C-peptide values were converted from pg/ml to pmol/l using a molar mass of 5808 g for insulin and 3010 g for C-peptide.

Plasma for the assessment of glucose was collected in NaF/KOx tubes on ice. Fasting and 120-min-post-load plasma glucose were measured in fresh samples with the enzymatic hexokinase method by use of two automatic analysers (i.e., the Beckman Synchron LX20 (Beckman Coulter Inc., USA) for samples obtained between November 2010 and April 2012, and the Roche Cobas 8000 (Roche Diagnostics, Mannheim, Germany) for samples obtained thereafter). Plasma for the assessment of glucose at other time points during the OGTT was separated after centrifugation (3000×*g* for 15 min at 4 °C), and stored within 2 h at − 80 °C until the assays were performed. Glucose was measured in these never-thawed samples with the enzymatic hexokinase method by use of the Roche Cobas 6000 (Roche Diagnostics, Mannheim, Germany). The Pearson correlation coefficient between fresh and frozen samples were 0.96 and 0.99, respectively, for fasting and 120-min-post-load plasma glucose samples (*n* = 486 samples).

#### β-Cell function and insulin sensitivity

As β-cell function consists of multiple components, it cannot be described by a single measure. Therefore, we used three mathematical model-based parameters (β-cell glucose sensitivity, potentiation factor ratio, and β-cell rate sensitivity) according to a previously described model [17], and two classic, relatively simple β-cell function indices (C-peptidogenic index and the ratio of the C-peptide to glucose area under the curve) [17–20]. These dynamic measures of BCF were included in the analyses, because they reflect both basal and post-load insulin secretory responses [11–13].

The mathematical model parameter ‘β-cell glucose sensitivity’ is the slope of the glucose–insulin secretion dose–response function [17], and represents the dependence of insulin secretion on absolute glucose concentration at any time point during the OGTT. β-Cell glucose sensitivity is a sensitive index to quantify β-cell dysfunction [21–23]. The dose–response relationship is modulated by β-cell potentiation, which accounts for higher insulin secretion during the descending phase of hyperglycemia than during the ascending phase of an OGTT, for the same glucose concentration. β-Cell potentiation is set as a positive function of time and averages 1 during the OGTT. Therefore, it represents the relative potentiation of the insulin secretion response to glucose. The β-cell potentiation parameter used in the present analysis represents the ratio of the β-cell potentiation factor at the end of the 2-h OGTT relative to the β-cell potentiation factor at the start. ‘β-Cell rate sensitivity’ is a marker of early phase insulin release, and represents the dynamic dependence of insulin secretion on the rate of change in glucose concentration [17].

The simple β-cell function indices C-peptidogenic index (ΔCP_30_/ΔG_30_) and the ratio of the C-peptide to glucose area under the curve (CP_AUC_/G_AUC_) were also calculated. The C-peptidogenic index (the equivalent of the insulinogenic index) reflects early phase insulin secretion and has good discriminatory ability to predict (pre)T2D (ROC AUCs ≥ 78%) [18]. The CP_AUC_/G_AUC_ is a measure of overall insulin secretion.

The Matsuda index (10,000/√*G*_0_ × *I*_0_ × mean *G* × mean *I*) was used as a measure of insulin sensitivity [24].

#### Dietary intake

All participants completed a validated 253-item food-frequency questionnaire (FFQ) [25] prior to being informed about their glucose metabolism status (e.g., normal glucose tolerance, prediabetes or T2D). Briefly, for each food item, frequency of consumption (ranging from ‘never or less than once a month’ to ‘every day’) and consumed amount were asked. Based on the FFQ data, daily intake of 23 main food product groups and nutrients were calculated using the Dutch Food Composition Database (NEVO) 2011 [26].

The FFQ was validated against a mean of 2.8 (range 1–5) 24-h recalls in 135 participants [25]. The correlation coefficient (95% CI) for beverage intake was 0.49 (0.44–0.76), and the FFQ ranked > 70% of the subjects correctly according to the beverage intake as compared to 24-h recalls [25].

The present study investigated consumption of artificially sweetened and sugar-sweetened soft drinks, juices, and total sugar-sweetened beverages, which were appraised by 20 items: artificially sweetened soft drink combines the information collected on consumption of artificially sweetened (non)carbonated lemonades and diluted syrups (2 items). Sugar-sweetened soft drinks incorporate sugar-sweetened (non)carbonated lemonades and diluted syrups (2 items). Juice combines the information collected on consumption of 100% or sugar-sweetened fruit and vegetable juices (5 items). Finally, total sugar-sweetened beverage combines sugar-sweetened soft drink, juices, nectars, energy drinks, sports drinks, and non-alcoholic beer (11 items). One serving is equivalent to 250 ml.

#### Anthropometric and other measurements

Body height (cm) and weight (kg) were measured to the nearest 1 cm and 0∙5 kg with the participants wearing light clothing and no shoes (Seca, Hamburg, Germany). Body mass index (BMI) was calculated as kilogram per meter squared (kg/m^2^). Blood pressure, and blood lipid profiles, including triglycerides, total cholesterol, HDL and LDL cholesterol, were determined as described previously [14]. Finally, smoking status (never, former, or current smoker), current medication use and history of cardiovascular disease (CVD) and cancer (yes/no) were self-reported. Moderate-to-vigorous physical activity (number of minutes/day with a step frequency > 110 step/min (~ 3.0 METs)) was measured using an activPAL3™ physical activity monitor (PAL Technologies, Glasgow, UK), as described elsewhere [27]. Detailed information concerning these measurements can be found in the study protocol of the Maastricht Study [14].

### Statistical analysis

All analyses were performed using the software package SPSS Statistics version 23.0 for Windows (SPSS, IBM Corp, Armonk, NY, USA).

Characteristics of the study population were described as means and standard deviations (SD) for continuous variables or as number and proportions of participants per category for categorical variables (% of study population). Differences across glucose metabolism groups were tested by the Kruskal–Wallis test. The Matsuda index was log-transformed to obtain normally distributed data.

Linear regression analyses were performed to assess associations of artificially sweetened and sugar-sweetened soft drinks, juice, and total sugar-sweetened beverage consumption with β-cell function indices (glucose sensitivity, potentiation factor ratio, C-peptidogenic index and overall insulin secretion) and insulin sensitivity measured by the Matsuda index, as continuous dependent variables. Logistic regression analyses were performed to assess the associations of the beverages with β-cell rate sensitivity, prediabetes, and T2D. β-Cell rate sensitivity was analyzed in tertiles (of which the highest tertile was considered the reference category), as the distribution of β-cell rate sensitivity was positively skewed and could not be normalized by transformations. Associations were presented as standardized regression coefficients [standardized betas (95% CI)] for continuous outcome measures and presented as odds ratios [OR (95% CI)] for categorical outcome measures.

Consumption of the beverages was analyzed categorically, comparing non-consumers with moderate (more than 1 consumption a month, less than daily consumption) and daily consumers.

To adjust for potential confounding, covariates were included in the regression models if their introduction in the model changed the regression coefficient of beverage consumption by > 10%. Furthermore, to assess β-cell function relative to the degree of insulin sensitivity, the Matsuda index was included as a covariate in all regression models with a β-cell function index as the dependent variable. Model 1 (crude model) was adjusted for sex and age. Model 2 was additionally adjusted for education level (low, middle, high), mean arterial blood pressure, antihypertensive medication, lipid-modifying medication, moderate-to-vigorous physical activity, HDL cholesterol, LDL cholesterol, triglycerides and intake of fibre, *trans* fat, red meat, and fruit. Furthermore, beverages were mutually adjusted. In our dataset, smoking status, CVD, family history of T2D, and the intake of saturated fat, alcohol, coffee, tea, dairy, vegetables, and sweets did not confound the associations of the individual mono- and disaccharides intake with β-cell function, insulin sensitivity, prediabetes and T2D and were, therefore, not included as covariates. The adjustments for total energy intake and BMI were performed in separate models (model 3 + total energy intake; and the fully adjusted model 4 + total energy intake + BMI) as both variables can act as confounders or as mediators in the associations of beverage consumption with glucose metabolism [28]. Whether or not BMI or total energy intake were confounders or mediators was investigated by use of the SPSS macro ‘PROCESS’ provided by Preacher and Hayes [29] in additional analyses.

## Results

### Population characteristics

Of the 2240 participants included in this study, 1420 (61.9%) individuals had normal glucose tolerance (NGT), 345 (15.6%) had prediabetes, 91 (3.9%) were newly diagnosed with T2D, and 384 (18.5%) were previously diagnosed with T2D (Table 1). The mean (SD) age was 59.5 (8.13) years and 50.4% were male.

**Table 1:** Characteristics of study population

	Total population	NGT	Prediabetes	ND T2D	PD T2D	*P* value*
*N*	2240	1420	345	91	384	
Sex (%male)	1130 (50.4)	625 (44.0)	183 (53.0)	61 (67.0)	261 (68.0)	< 0.01
Age	59.5 ± 8.13	58.0 ± 8.01	61.5 ± 7.46	63.9 ± 7.21	62.3 ± 7.70	< 0.01
BMI category (kg/m^2^)						< 0.01
< 25	840 (37.5)	680 (47.5)	88 (25.5)	18 (19.8)	54 (14.1)	
25–30	972 (43.4)	587 (41.3)	160 (46.4)	45 (49.5)	180 (46.9)	
≥ 30	428 (19.1)	153 (10.8)	97 (28.1)	28 (30.8)	150 (39.1)	
Smoking						0.03
Never (%)	828 (37.0)	572 (40.3)	107 (31.0)	34 (37.4)	115 (30.2)	
Former (%)	1158 (51.8)	683 (48.3)	204 (59.1)	47 (51.7)	224 (58.8)	
Current (%)	250 (11.2)	164 (11.6)	34 (9.9)	11 (11.0)	42 (11.0)	
Systolic blood pressure (mmHg)	134 ± 17.8	131 ± 17.2	137 ± 16.5	145 ± 18.7	141 ± 17.1	< 0.01
Diastolic blood pressure (mmHg)	76.4 ± 9.92	75.3 ± 9.88	78.1 ± 9.87	79.0 ± 10.3	78.2 ± 9.47	< 0.01
CVD (%yes)	280 (14.7)	164 (11.7)	40 (11.7)	24 (26.4)	87 (23.3)	< 0.01
Total PA (h/week)	14.2 ± 7.91	14.8 ± 7.90	14.3 ± 7.64	13.2 ± 7.24	12.4 ± 8.08	< 0.01
MVPA (h/week)	5.59 ± 4.20	6.05 ± 4.22	5.17 ± 4.02	4.89 ± 3.86	4.41 ± 4.10	< 0.01
Total energy intake (kcal)	2187 ± 597	2200 ± 593	2195 ± 580	2169 ± 634	2137 ± 619	0.18
Artificially sweetened soft drink (g/day^a^)	42.9 ± 108	35.5 ± 98.2	42.7 ± 109	40.5 ± 123	71.0 ± 131	< 0.01
Sugar-sweetened soft drink (g/day^a^)	36.1 ± 86.5	34.5 ± 84.4	33.3 ± 69.2	49.3 ± 132	41.4 ± 94.1	0.22
Juice (g/day^a^)	56.8 ± 80.4	59.1 ± 81.6	56.4 ± 76.4	58.7 ± 85.5	48.1 ± 78.1	< 0.01
Total sugar-sweetened beverage (g/day)	119 ± 151	118 ± 149	115 ± 138	128 ± 178	121 ± 164	0.23
HbA1c (%)	5.72 ± 0.63	5.44 ± 0.35	5.70 ± 0.40	6.21 ± 0.61	6.67 ± 0.62	< 0.01
HDL:LDL ratio	0.55 ± 0.27	0.54 ± 0.35	0.51 ± 0.23	0.48 ± 0.20	0.61 ± 0.29	< 0.01
Triglycerides (mmol/l)	1.40 ± 0.84	1.23 ± 0.67	1.61 ± 1.08	1.99 ± 1.13	1.69 ± 0.88	< 0.01
Β-cell glucose sensitivity (pmol/min/m^2^/mM)	27.8 ± 18.2	33.3 ± 18.7	26.1 ± 12.4	17.1 ± 10.8	11.4 ± 8.03	< 0.01
Β-cell rate sensitivity (pmol/m^2^/mM)	250 ± 312	302 ± 350	238 ± 230	190 ± 255	82.5 ± 107	< 0.01
Β-cell potentiation factor	1.65 ± 0.70	1.82 ± 0.75	1.54 ± 0.55	1.41 ± 0.44	1.16 ± 0.34	< 0.01
Overall insulin secretion (CP_AUC_:G_AUC_ ratio)	196 ± 77.7	217 ± 71.0	197 ± 75.1	159 ± 70.6	125 ± 58.7	< 0.01
C-peptidogenic index (∆CP_30_:∆G_30_ ratio)	460 ± 1178	597 ± 1414	310 ± 752	247 ± 207	144 ± 116	< 0.01
Matsuda index	3.98 ± 2.63	4.77 ± 2.67	2.87 ± 1.97	2.16 ± 1.37	2.49 ± 1.86	< 0.01

*BMI* Body Mass Index, *NGT* normal glucose tolerance, *ND T2D* newly diagnosed type 2 diabetes mellitus, *PD* previously diagnosed type 2 diabetes mellitus, *CVD* cardiovascular disease, *PA* physical activity, *MVPA* moderate-to-vigorous physical activity of total waking time, *HbA1C* glycated haemoglobin, *HDL* high-density lipoprotein, *LDL* low-density lipoprotein

*P value of Kruskal–Wallis test

^a^g/day is equal to ml/day

Of the participants excluded from analyses (*n* = 885, see “Methods” section for reasons), mean (SD) age was 59.9 (8.60) years and 53% were male; these characteristics were essentially the same as the current study population. The proportion of individuals previously diagnosed with T2DM was higher than in the current population, which is mainly due to the use of insulin treatment (*n* = 220) and according to protocol no OGTT could be performed.

The prevalence of obesity, hypertension, and history of CVD was lowest among NGT individuals and highest among T2D individuals. Previously diagnosed T2D persons had the highest HDL:LDL ratio, most likely due to lipid-modifying medication. Indices for BCF and insulin sensitivity were highest among NGT individuals (representing good BCF and insulin sensitivity) and lowest among T2D individuals (representing impaired BCF and insulin resistance). Moreover, self-reported artificially sweetened soft drink consumption was lowest in the NGT group and highest in previously diagnosed T2D persons. Total juice consumption was highest in the NGT group and lowest in the previously diagnosed T2D persons. Sugar-sweetened soft drink and total sugar-sweetened beverage consumption were comparable between the glucose metabolism groups (Table 1). Overall, the consumption of artificially sweetened and sugar-sweetened beverages was relatively low. The median beverage intake of the moderate and daily consumers groups are shown in Table 2.

**Table 2:** Median beverage intake

	Moderate Median (25th–75th percentage)	Daily Median (25th–75th percentage)
Artificially sweetened soft drink consumption (g/day^a^)	12.0 (1.80–60.0)	300 (300–450)
Sugar-sweetened soft drink consumption (g/day^a^)	12.0 (1.80–42.8)	300 (259–386)
Juice consumption (g/day^a^)	24.0 (9.00–85.5)	300 (216–320)
Total sugar-sweetened drink consumption (g/day^a^)	48.9 (17.4–110.9)	318 (260–434)

^a^g/day is equal to ml/day

Daily consumers of artificially sweetened soft drinks and non-consumers of juice were more often previously diagnosed with T2D and less often normoglycemic. Furthermore, daily sugar-sweetened soft drink consumers, and moderate and daily artificially sweetened soft drink consumers had higher prevalence of overweight/obesity compared to the non-consumer groups (data not shown).

### Associations of artificially sweetened soft drink consumption with BCF and insulin sensitivity

Both moderate and daily vs. no artificially sweetened soft drink consumption was significantly associated with lower β-cell glucose sensitivity, β-cell potentiation, total insulin secretion, insulin sensitivity (as measured by the Matsuda index) (Table 3) and β-cell rate sensitivity (Table 4) in the crude model.

**Table 3:** Associations of artificially and sugar-sweetened beverage consumption with continuous BCF and insulin sensitivity measures (standardized betas and 95% confidence intervals)

	Model	Non-consumers (ref)	Moderate consumers	Daily consumers
		*Β*	*β* (95% CI)	*β* (95% CI)
Artificially sweetened soft drink				
*N*		1187	887	166
β-Cell glucose sensitivity	Crude	0	− 0.08 (− 0.12, − 0.04)	− 0.08 (− 0.12, − 0.03)
	Fully adjusted	0	− 0.06 (− 0.11, − 0.02)	− 0.05 (− 0.09, 0.00)
β-Cell potentiation factor	Crude	0	− 0.04 (− 0.08, 0.01)	− 0.06 (− 0.10, − 0.01)
	Fully adjusted	0	− 0.02 (− 0.06, 0.03)	− 0.02 (− 0.06, 0.02)
C-peptidogenic index	Crude	0	− 0.02 (− 0.06, 0.02)	− 0.01 (− 0.06, 0.03)
	Fully adjusted	0	− 0.02 (− 0.06, 0.03)	− 0.01 (− 0.05, 0.04)
Overall insulin secretion	Crude	0	− 0.07 (− 0.11, − 0.03)	− 0.07 (− 0.11, − 0.03)
	Fully adjusted	0	− 0.06 (− 0.10, − 0.02)	− 0.05 (− 0.09, − 0.01)
Matsuda index^a^	Crude	0	− 0.06 (− 0.10, − 0.02)	− 0.09 (− 0.13, − 0.05)
	Fully adjusted	0	− 0.01 (− 0.05, 0.03)	− 0.01 (− 0.05, 0.03)
Sugar-sweetened soft drink				
*N*		825	1299	116
β-Cell glucose sensitivity	Crude	0	0.03 (− 0.02, 0.07)	− 0.01 (− 0.06, 0.03)
	Fully adjusted	0	0.00 (− 0.04, 0.05)	− 0.01 (− 0.06, 0.03)
β-Cell potentiation factor	Crude	0	0.02 (− 0.02, 0.06)	− 0.03 (− 0.07, 0.02)
	Fully adjusted	0	0.01 (− 0.04, 0.05)	− 0.02 (− 0.07, 0.02)
C-peptidogenic index	Crude	0	0.02 (− 0.03, 0.06)	− 0.02 (− 0.06, 0.03)
	Fully adjusted	0	0.01 (− 0.03, 0.06)	− 0.02 (− 0.06, 0.03)
Overall insulin secretion	Crude	0	0.03 (− 0.02, 0.07)	− 0.01 (− 0.05, 0.04)
	Fully adjusted	0	0.01 (− 0.05, 0.04)	− 0.01 (− 0.05, 0.03)
Matsuda index^a^	Crude	0	0.00 (− 0.04, 0.04)	− 0.04 (− 0.08, 0.01)
	Fully adjusted	0	0.00 (− 0.04, 0.03)	− 0.01 (− 0.05, 0.03)
Juice				
*N*		274	1851	115
β-Cell glucose sensitivity	Crude	0	0.06 (0.01, 0.11)	0.02 (− 0.03, 0.07)
	Fully adjusted	0	0.03 (− 0.02, 0.08)	0.00 (− 0.05, 0.05)
β-Cell potentiation factor	Crude	0	0.01 (− 0.04, 0.05)	0.02 (− 0.02, 0.07)
	Fully adjusted	0	− 0.02 (− 0.06, 0.03)	0.02 (− 0.03, 0.06)
C-peptidogenic index	Crude	0	0.01 (− 0.04, 0.06)	0.00 (− 0.05, 0.05)
	Fully adjusted	0	0.01 (− 0.04, 0.06)	0.00 (− 0.05, 0.05)
Overall insulin secretion	Crude	0	0.05 (0.1, 0.10)	0.02 (− 0.03, 0.06)
	Fully adjusted	0	0.02 (− 0.02, 0.07)	− 0.01 (− 0.06, 0.04)
Matsuda index^a^	Crude	0	0.06 (0.02, 0.11)	0.01 (− 0.03, 0.06)
	Fully adjusted	0	0.03 (− 0.02, 0.07)	0.01 (− 0.04, 0.05)
Total sugar-sweetened beverage				
*N*		145	1689	406
β-Cell glucose sensitivity	Crude	0	0.08 (0.01, 0.16)	0.05 (− 0.03, 0.12)
	Fully adjusted	0	0.05 (− 0.02, 0.12)	0.02 (− 0.05, 0.09)
β-Cell potentiation factor	Crude	0	0.04 (− 0.03, 0.11)	0.01 (− 0.06, 0.09)
	Fully adjusted	0	0.02 (− 0.06, 0.09)	0.01 (− 0.06, 0.08)
C-peptidogenic index	Crude	0	0.03 (− 0.04, 0.11)	0.01 (− 0.07, 0.08)
	Fully adjusted	0	0.03 (− 0.05, 0.10)	0.01 (− 0.07, 0.09)
Overall insulin secretion	Crude	0	0.08 (0.01, 0.15)	0.03 (− 0.04, 0.10)
	Fully adjusted	0	0.04 (− 0.03, 0.11)	− 0.01 (− 0.08, 0.07)
Matsuda index^a^	Crude	0	0.08 (0.01, 0.15)	0.00 (− 0.07, 0.08)
	Fully adjusted	0	0.04 (− 0.03, 0.10)	0.01 (− 0.05, 0.08)

Positive values indicate a better BCF or insulin sensitivity, and negative values indicate a lower BCF or insulin sensitivity

Crude model: model adjusted for sex, age, and insulin sensitivity

Fully adjusted model: model adjusted for sex, age, insulin sensitivity, education, blood pressure expressed in MAP, lipid-modifying medication, antihypertensive medication, moderate-to-vigorous physical activity, high-density cholesterol, low-density cholesterol, triglycerides, intake of dietary fibre, *trans* fat, red meat and fruit intake, mutual adjustment for the other beverage category, total energy intake, and body mass index

^a^Matsuda index is not adjusted for insulin sensitivity

**Table 4:** Associations of artificially and sugar-sweetened beverage intake with β-cell rate sensitivity tertiles (odds ratios and 95% confidence intervals)

	Tertile 1 vs. 3 β-cell rate sensitivity			
		Non-consumers (ref)	Moderate consumers	Daily consumers
	Model	OR	OR (95% CI)	OR (95% CI)
Artificially sweetened soft drink				
*N*		1187	887	166
	Crude	1	1.35 (1.09, 1.69)	1.54 (1.09, 1.69)
	Fully adjusted	1	1.29 (1.03, 1.62)	1.26 (0.83, 1.91)
Sugar-sweetened soft drink				
*N*		825	1299	116
	Crude	1	0.90 (0.72, 1.12)	1.47 (0.91, 2.38)
	Fully adjusted	1	0.95 (0.75, 1.21)	1.46 (0.87, 2.43)
Juice				
*N*		274	1851	115
	Crude	1	0.69 (0.50, 0.95)	0.59 (0.35, 1.02)
	Fully adjusted	1	0.82 (0.59, 1.15)	0.72 (0.41, 1.27)
Total sugar-sweetened beverage				
*N*		145	1689	406
	Crude	1	0.66 (0.43, 1.01)	0.85 (0.53, 1.38)
	Fully adjusted	1	0.77 (0.50, 1.19)	0.99 (0.60, 1.63)

Third tertile of rate sensitivity is the reference group (best rate sensitivity)

Values < 1.00 indicate a better β-cell rate sensitivity, and values > 1.00 indicate a lower β-cell rate sensitivity

Crude model: model adjusted for sex, age, and insulin sensitivity

Fully adjusted model: model adjusted for sex, age, insulin sensitivity, education, blood pressure expressed in MAP, lipid-modifying medication, antihypertensive medication, moderate-to-vigorous physical activity, high-density cholesterol, low-density cholesterol, triglycerides, intake of dietary fibre, *trans* fat, red meat and fruit intake, mutual adjustment for the other beverage category, total energy intake, and body mass index

In the fully adjusted model, artificially sweetened soft drink consumption was significantly associated with lower β-cell glucose sensitivity, with beta (95% CI) of − 0.06 (− 0.11, − 0.02) for moderate consumption, and − 0.05 (− 0.09, − 0.00) for daily consumption, respectively. Similar associations were observed between artificially sweetened soft drink consumption and lower overall insulin secretion, with beta (95% CI) of − 0.06 (− 0.10, − 0.02) for moderate consumption, and of − 0.05 (− 0.09, − 0.01) for daily consumption, respectively (Table 3). Moderate, but not daily consumption, was associated with β-cell rate sensitivity compared to abstainers, with OR (95% CI) of 1.29 (1.03, 1.62) (Table 4). No associations were observed for artificially sweetened soft drink consumption with prediabetes and diabetes (see Online Appendix Tables 1 and 2).

Associations remained essentially the same after exclusion of participants who were previously diagnosed with T2DM.

### Associations of sugar-sweetened soft drink, juice and total sugar-sweetened beverage consumption with BCF and insulin sensitivity

Moderate compared to abstainers of total sugar-sweetened drink consumption was associated with higher β-cell glucose sensitivity, higher overall insulin secretion and higher insulin sensitivity in the crude models (Table 3). None of the associations remained significant after further adjustments.

No significant associations were observed for moderate and daily consumption compared with no consumption of sugar-sweetened soft drink (Tables 3, 4; Online Appendix Tables 1 and 2).

Associations remained essentially the same after exclusion of participants who were previously diagnosed with T2DM.

### Additional analyses

We additionally investigated whether total energy intake and BMI acted as confounders, effect modifiers or mediators in the association of beverage consumption with glucose metabolism. Total energy intake and BMI did not act as main confounders, as the associations remained essentially the same after additional adjustments for total energy intake and BMI (see Online Appendix Tables 3 and 4).

Furthermore, having a normal weight, overweight or obesity did not significantly modify the associations. Finally, mediation analyses revealed no mediating effects of total energy intake nor of BMI in the associations of beverage consumption with BCF (data not shown).

## Discussion

The novelty of this study is the evaluation of the associations of artificially sweetened and sugar-sweetened soft drink, juice and total sugar-sweetened beverage consumption with multiple dynamic indices of pancreatic BCF and insulin sensitivity. After adjustment for confounders, artificially sweetened soft drink consumption was associated with lower β-cell glucose sensitivity, overall insulin secretion, and β-cell rate sensitivity. No associations were observed for the consumption of sugar-sweetened soft drinks, juices and total sugar-sweetened beverages with BCF, insulin sensitivity, and glucose metabolism status.

In our analyses, artificially sweetened soft drink consumption was associated with lower β-cell glucose sensitivity, β-cell rate sensitivity, and overall insulin secretion. These findings are in line with one previous observational study that showed an association of artificially sweetened beverage consumption with a lower fasting BCF and lower insulin sensitivity in lean participants (BMI < 25 kg/m^2^) only [6], but not with three other observational studies who did not show an association with insulin sensitivity [4, 5, 7]. These discrepant findings may be caused by differences in research methodology, i.e., three studies focused on fasting insulin sensitivity only [4–6] and two studies excluded newly diagnosed T2D from analyses [5, 7]. In addition, however, randomized controlled trials so far have not been able to detect any effects of artificial sweeteners on glucose metabolism [30, 31]. Therefore, future studies are necessary to confirm our results.

In a meta-analysis of prospective observational studies [9], all except one study showed that a higher artificially sweetened beverage consumption was significantly [9, 28, 32–36] or weakly (nonsignificant) associated [37, 38] with higher T2D risk in analyses without adjustment for adiposity. After additional adjustment for adiposity, associations were attenuated towards the null in four of these studies [9, 28, 32, 34]. In line with these studies, high consumption of artificially sweetened soft drinks was positively, but non-significantly, associated with prediabetes and T2D in the present study. The observed associations of higher artificially sweetened soft drink consumption with lower BCF indicate an association between higher artificially sweetened soft drink consumption and impaired glucose homeostasis. However, the range of exposure to artificially sweetened soft drink consumption varied from 0 to 300 g/day (percentiles 5–95) in the current study population, and this range might be too small to detect significant associations with prediabetes and T2D because of loss in information when using binary outcome measures.

The mechanisms by which artificially sweetened beverage consumption may modify glucose homeostasis are not fully understood. One suggested mechanism is that artificially sweeteners alter gut microbiota resulting in impaired glucose homeostasis [6, 39, 40]. Other studies suggest that artificially sweeteners increase the hedonistic desire for sweet taste [33, 41], an overestimation of beverage calories saved by consuming non-caloric instead of caloric beverages [33, 34], and/or increased appetite [4], which all might result in a higher total energy intake and weight gain, and subsequently a higher risk of disturbed glucose homeostasis. However, mediation analyses in the present data suggest that an increase in energy intake and BMI is not the main pathway by which artificially sweetened soft drink consumption causes impaired glucose homeostasis. Others argue that the observed association is mainly caused by reversed causality [32, 42, 43]. This hypothesis implies that persons started to consume artificially sweetened beverages in an attempt to lose weight or to better control glucose homeostasis after being diagnosed with T2D. However, sensitivity analyses in another study [36] and the present study indicate that reverse causality might not explain the association of a higher consumption of artificially sweetened beverage consumption with a higher risk of disturbed glucose homeostasis. As in our study, the associations of artificially sweetened soft drink consumption with BCF remained significant after exclusion of participants who were previously diagnosed with T2D. Moreover, BMI did not modify the association between artificially sweetened soft drink consumption and measures of glucose homeostasis.

The absence of an association of total sugar-sweetened beverage consumption and sugar-sweetened soft drinks with measures of glucose homeostasis are in line with studies focusing on T2D as an outcome [7, 30, 33, 44], but in contrast with others who found that higher consumption of total sugar-sweetened and sugar-sweetened soft drinks was positively associated with T2D [28, 34, 37, 45]. In one study [37] the positive association attenuated towards the null after adjustment for BMI though. Studies that focused on fasting measures of insulin sensitivity even showed an inverse association with total sugar-sweetened beverage consumption [4, 5, 7]. These conflicting findings could be partly due to differences in consumption of sugar-sweetened beverages between study populations (which was relatively low in ours), or in beverages included in the sugar-sweetened beverage category [7, 9]. In addition, fasting (static) measures of insulin sensitivity are less reliable than dynamic indices of insulin sensitivity [46].

In our study, we did not observe associations of juice consumption with β-cell function, insulin sensitivity or glucose metabolism status. A recent meta-analysis [47] showed that 100% juices were not associated with T2D, whereas sugar-sweetened juices were positively associated with T2D [47]. As our data did not allow dissecting between consumption of 100% juices and sugar-sweetened fruit- and vegetable-based juices, no conclusions regarding the association of juices with BCF and insulin sensitivity can be drawn.

One of the strengths of our study is that β-cell dysfunction and lower insulin sensitivity provide physiological information on early abnormalities in glucose metabolism that eventually result in prediabetes and T2D. Furthermore, as beta-cell function is a continuous outcome measure it has a larger statistical power to detect associations compared to the dichotomous outcome measure T2D. Another strength is the use of multiple indices to assess various dynamic aspects of BCF and insulin sensitivity indices which better reflect metabolic conditions. In addition, the large sample size and the inclusion of participants covering the total glucose metabolism spectrum from NGT to T2D, and the extensive data collection on demographics and lifestyle factors, allowed a thorough adjustment for confounders. One limitation is the cross-sectional design of our study, by which causality could not be determined. Furthermore, there is a risk of reporting/information bias; however, as all participants completed the FFQ prior to being informed about their glucose metabolism status, we consider the risk of reporting bias is minimal. Finally, the degree of variation in consumption of sugar-sweetened soft drinks, juice and total sugar-sweetened beverage consumption may have been somewhat low to estimate associations with measures of glucose homeostasis.

## Conclusions

In the current study, artificially sweetened soft drink consumption was associated with lower BCF. We found no evidence for associations of sugar-sweetened soft drinks, juices and total sugar-sweetened beverages with BCF, insulin sensitivity and glucose metabolism status. This is the first study on the association of artificially and sugar-sweetened soft drink, juice and total sugar-sweetened beverage consumption with non-fasting insulin sensitivity and multiple BCF aspects. Therefore, prospective studies are warranted to confirm current findings and further investigate the role of ASB and SSB in observational and clinical settings.

## Electronic supplementary material

Below is the link to the electronic supplementary material.

Supplementary material 1 (DOCX 37 kb)
