# Supplementary material for: Association of artificially sweetened and sugar-sweetened soft drinks with β-cell function, insulin sensitivity, and type 2 diabetes: the Maastricht Study
Source: Eur J Nutr. 2019 Sep 5;59(4):1717–27. doi: 10.1007/s00394-019-02026-0 (PMC7230048; doi:10.1007/s00394-019-02026-0)
Supplement: Supplementary file 1 — Supplementary material 1 (DOCX 37 kb) [file 394_2019_2026_MOESM1_ESM.docx]

| **Appendix Table 1:** Associations of artificially and sugar-sweetened beverage intake with prediabetes (odds ratios and 95% confidence intervals) | | | | |
| --- | --- | --- | --- | --- |
|  | **Tertile 1 versus 3 β-cell rate sensitivity** | | | |
|  |  | **Non consumers**  **(ref)** | **Moderate consumers** | **Daily consumers** |
|  | Model | OR | OR (95%CI) | OR (95%CI) |
| **Artificially sweetened soft drink** | *N* | *632* | *1049* | *74* |
|  | Crude | 1 | 1.30 (1.01, 1.68) | 1.61 (0.93, 2.79) |
|  | Fully adjusted | 1 | 1.19 (0.88, 1.62) | 1.01 (0.49, 2.08) |
| **Sugar-sweetened soft drink** | *N* | *632* | *1049* | *74* |
|  | Crude | 1 | 0.96 (0.74, 1.23) | 0.88 (0.46, 1.68) |
|  | Fully adjusted | 1 | 0.82 (0.60, 1.12) | 0.58 (0.25, 1.33) |
| **Total sugar-sweetened beverage** | *N* | *965* | *709* | *91* |
|  | Crude | 1 | 0.63 (0.39, 1.02) | 0.90 (0.51, 1.56) |
|  | Fully adjusted | 1 | 0.72 (0.40, 1.30) | 0.68 (0.34, 1.40) |
| Fully adjusted model: model adjusted for sex, age, education, blood pressure expressed in MAP, lipid-modifying medication, antihypertensive medication, moderate-to-vigorous physical activity, high-density cholesterol, low-density cholesterol, triglycerides, intake of dietary fibre, *trans* fat, red meat and fruit intake, mutual adjustment for the other beverage category, total energy intake and body mass index | | | | |

| **Appendix Table 2:** Associations of artificially and sugar-sweetened beverage intake with diabetes type 2 (odds ratios and 95% confidence intervals) | | | | |
| --- | --- | --- | --- | --- |
|  | **Tertile 1 versus 3 β-cell rate sensitivity** | | | |
|  |  | **Non consumers**  **(ref)** | **Moderate consumers** | **Daily consumers** |
|  | Model | OR | OR (95%CI) | OR (95%CI) |
| **Artificially sweetened soft drink** | N | *1026* | *733* | *97* |
|  | Crude | 1 | 0.65 (0.40, 1.07) | 1.60 (0.65, 3.92) |
|  | Fully adjusted | 1 | 0.71 (0.40, 1.25) | 1.18 (0.40, 3.45) |
| **Sugar-sweetened soft drink** |  | 93 | 1419 | 253 |
|  | Crude | 1 | 1.45 (0.90, 2.35) | 2.09 (0.75, 5.81) |
|  | Fully adjusted | 1 | 1.18 (0.66, 2.12) | 0.91 (0.18, 4.56) |
| **Total sugar-sweetened beverage** |  | *632* | *1049* | *74* |
|  | Crude | 1 | 0.93 (0.38, 2.24) | 1.36 (0.50, 3.67) |
|  | Fully adjusted | 1 | 0.83 (0.32, 2.18) | 1.07 (0.34, 3.41) |
| Fully adjusted model: model adjusted for sex, age, education, blood pressure expressed in MAP, lipid-modifying medication, antihypertensive medication, moderate-to-vigorous physical activity, high-density cholesterol, low-density cholesterol, triglycerides, intake of dietary fibre, *trans* fat, red meat and fruit intake, mutual adjustment for the other beverage category, total energy intake and body mass index | | | | |

| **Appendix Table 3:** Associations of artificially and sugar-sweetened beverage consumption with continuous BCF and insulin sensitivity measures (standardized betas and 95% confidence intervals) | | | | |
| --- | --- | --- | --- | --- |
|  |  | Non consumers  (ref) | Moderate consumers | Daily consumers |
|  | Model | Β | β (95%CI) | β (95%CI) |
| **Artificially sweetened soft drink** |  |  |  |  |
| N |  | *1187* | *887* | *166* |
| β-cell glucose sensitivity | 1 | 0 | -0.08 (-0.12, -0.04) | -0.08 (-0.12, -0.03) |
|  | 2 | 0 | -0.07 (-0.11, -0.03) | -0.05 (-0.09, -0.01) |
|  | 3 | 0 | -0.07 (-0.11, -0.03) | -0.05 (-0.09, -0.01) |
|  | 4 | 0 | -0.06 (-0.11, -0.02) | -0.05 (-0.09, 0.00) |
| β-cell potentiation factor | 1 | 0 | -0.04 (-0.08, 0.01) | -0.06 (-0.10, -0.01) |
|  | 2 | 0 | -0.02 (-0.07, 0.02) | -0.03 (-0.07, 0.02) |
|  | 3 | 0 | -0.02 (-0.07, 0.02) | -0.03 (-0.07, 0.01) |
|  | 4 | 0 | -0.02 (-0.06, 0.03) | -0.02 (-0.06, 0.02) |
| C-peptidogenic index | 1 | 0 | -0.02 (-0.06, 0.02) | -0.01 (-0.06, 0.03) |
|  | 2 | 0 | -0.02 (-0.06, 0.03) | -0.01 (-0.05, 0.04) |
|  | 3 | 0 | -0.02 (-0.06, 0.03) | -0.01 (-0.05, 0.04) |
|  | 4 | 0 | -0.02 (-0.06, 0.03) | -0.01 (-0.05, 0.04) |
| Overall insulin secretion | 1 | 0 | -0.07 (-0.11, -0.03) | -0.07 (-0.11, -0.03) |
|  | 2 | 0 | -0.06 (-0.10, -0.02) | -0.05 (-0.09, -0.01) |
|  | 3 | 0 | -0.06 (-0.10, -0.02) | -0.05 (-0.09, -0.01) |
|  | 4 | 0 | -0.06 (-0.10, -0.02) | -0.05 (-0.09, -0.01) |
| Matsuda index * | 1 | 0 | -0.06 (-0.10, -0.02) | -0.09 (-0.13, -0.05) |
|  | 2 | 0 | -0.04 (-0.08, 0.00) | -0.04 (-0.08, 0.00) |
|  | 3 | 0 | -0.04 (-0.08, 0.00) | -0.04 (-0.08, 0.00) |
|  | 4 | 0 | -0.01 (-0.05, 0.03) | -0.01 (-0.05, 0.03) |
|  |  |  |  |  |
| **Sugar-sweetened**  **soft drink** |  |  |  |  |
| N |  | *825* | 1299 | *134* |
| β-cell glucose sensitivity | 1 | 0 | 0.03 (-0.02, 0.07) | -0.01 (-0.06, 0.03) |
|  | 2 | 0 | 0.01 (-0.04, 0.05) | -0.01 (-0.06, 0.03) |
|  | 3 | 0 | 0.00 (-0.04, 0.05) | -0.01 (-0.06, 0.03) |
|  | 4 | 0 | 0.00 (-0.04, 0.05) | -0.01 (-0.06, 0.03) |
| β-cell potentiation factor | 1 | 0 | 0.02 (-0.02, 0.06) | -0.03 (-0.07, 0.02) |
|  | 2 | 0 | 0.01 (-0.03, 0.06) | -0.02 (-0.07, 0.02) |
|  | 3 | 0 | 0.01 (-0.03, 0.06) | -0.02 (-0.07, 0.02) |
|  | 4 | 0 | 0.01 (-0.04, 0.05) | -0.02 (-0.07, 0.02) |
| C-peptidogenic index | 1 | 0 | 0.02 (-0.03, 0.06) | -0.02 (-0.06, 0.03) |
|  | 2 | 0 | 0.01 (-0.03, 0.06) | -0.02 (-0.06, 0.03) |
|  | 3 | 0 | 0.01 (-0.03, 0.06) | -0.02 (-0.06, 0.03) |
|  | 4 | 0 | 0.01 (-0.03, 0.06) | -0.02 (-0.06, 0.03) |
| Overall insulin secretion | 1 | 0 | 0.03 (-0.02, 0.07) | -0.01 (-0.05,0.04) |
|  | 2 | 0 | 0.01 (-0.05, 0.04) | -0.01 (-0.05, 0.03) |
|  | 3 | 0 | 0.01 (-0.05, 0.04) | -0.01 (-0.05, 0.03) |
|  | 4 | 0 | 0.01 (-0.05, 0.04) | -0.01 (-0.05, 0.03) |
| Matsuda index * | 1 | 0 | 0.00 (-0.04, 0.04) | -0.04 (-0.08, 0.01) |
|  | 2 | 0 | 0.00 (-0.04, 0.03) | -0.01 (-0.05, 0.04) |
|  | 3 | 0 | 0.00 (-0.04, 0.03) | -0.01 (-0.05, 0.04) |
|  | 4 | 0 | 0.00 (-0.04, 0.03) | -0.01 (-0.05, 0.03) |
| **Juice** |  |  |  |  |
| N |  | *274* | *1851* | *370* |
| β-cell glucose sensitivity | 1 | 0 | 0.06 (0.01, 0.11) | 0.02 (-0.03, 0.07) |
|  | 2 | 0 | 0.03 (-0.02, 0.08) | 0.00 (-0.05, 0.05) |
|  | 3 | 0 | 0.03 (-0.02, 0.08) | 0.00 (-0.05, 0.05) |
|  | 4 | 0 | 0.03 (-0.02, 0.08) | 0.00 (-0.05, 0.05) |
| β-cell potentiation factor | 1 | 0 | 0.01 (-0.04, 0.05) | 0.02 (-0.02, 0.07) |
|  | 2 | 0 | -0.02 (-0.06, 0.03) | 0.02 (-0.03, 0.06) |
|  | 3 | 0 | -0.02 (-0.06, 0.03) | 0.02 (-0.03, 0.06) |
|  | 4 | 0 | -0.02 (-0.06, 0.03) | 0.02 (-0.03, 0.06) |
| C-peptidogenic index | 1 | 0 | 0.01 (-0.04, 0.06) | 0.00 (-0.05, 0.05) |
|  | 2 | 0 | 0.01 (-0.04,0.06) | 0.00 (-0.05, 0.05) |
|  | 3 | 0 | 0.01 (-0.04,0.06) | 0.00 (-0.05, 0.05) |
|  | 4 | 0 | 0.01 (-0.04,0.06) | 0.00 (-0.05, 0.05) |
| Overall insulin secretion | 1 | 0 | 0.05 (0.1, 0.10) | 0.02 (-0.03, 0.06) |
|  | 2 | 0 | 0.02 (-0.02, 0.07) | -0.01 (-0.06, 0.04) |
|  | 3 | 0 | 0.02 (-0.02, 0.07) | -0.01 (-0.06, 0.04) |
|  | 4 | 0 | 0.02 (-0.02, 0.07) | -0.01 (-0.06, 0.04) |
| Matsuda index * | 1 | 0 | 0.06 (0.02, 0.11) | 0.01 (-0.03, 0.06) |
|  | 2 | 0 | 0.03 (-0.02, 0.07) | 0.01 (-0.03, 0.06) |
|  | 3 | 0 | 0.03 (-0.02, 0.07) | 0.01 (-0.03, 0.06) |
|  | 4 | 0 | 0.03 (-0.02, 0.07) | 0.01 (-0.04, 0.05) |
| **Total sugar-sweetened beverage** |  |  |  |  |
| N |  | *145* | *1689* | *471* |
| β-cell glucose sensitivity | 1 | 0 | 0.08 (0.01, 0.16) | 0.05 (-0.03, 0.12) |
|  | 2 | 0 | 0.05(-0.02, 0.12) | 0.02 (-0.06, 0.09) |
|  | 3 | 0 | 0.05(-0.02, 0.12) | 0.02 (-0.06, 0.09) |
|  | 4 | 0 | 0.05(-0.02, 0.12) | 0.02 (-0.05, 0.09) |
| β-cell potentiation factor | 1 | 0 | 0.04 (-0.03, 0.11) | 0.01 (-0.06, 0.09) |
|  | 2 | 0 | 0.02 (-0.06, 0.09) | 0.01 (-0.06, 0.08) |
|  | 3 | 0 | 0.02 (-0.06, 0.09) | 0.01 (-0.06, 0.08) |
|  | 4 | 0 | 0.02 (-0.06, 0.09) | 0.01 (-0.06, 0.08) |
| C-peptidogenic index | 1 | 0 | 0.03 (-0.04, 0.11) | 0.01 (-0.07, 0.08) |
|  | 2 | 0 | 0.03 (-0.05, 0.10) | 0.01 (-0.07, 0.08) |
|  | 3 | 0 | 0.03 (-0.05, 0.10) | 0.01 (-0.07, 0.08) |
|  | 4 | 0 | 0.03 (-0.05, 0.10) | 0.01 (-0.07, 0.09) |
| Overall insulin secretion | 1 | 0 | 0.08 (0.01, 0.15) | 0.03 (-0.04, 0.10) |
|  | 2 | 0 | 0.04 (-0.03, 0.11) | 0.00 (-0.07, 0.07) |
|  | 3 | 0 | 0.04 (-0.03, 0.11) | -0.01 (-0.08, 0.07) |
|  | 4 | 0 | 0.04 (-0.03, 0.11) | -0.01 (-0.08, 0.07) |
| Matsuda index * | 1 | 0 | 0.08 (0.01, 0.15) | 0.00 (-0.07, 0.08) |
|  | 2 | 0 | 0.04 (-0.02, 0.11) | 0.02 (-0.05, 0.09) |
|  | 3 | 0 | 0.04 (-0.02, 0.11) | 0.01 (-0.05, 0.08) |
|  | 4 | 0 | 0.04 (-0.03, 0.10) | 0.01 (-0.05, 0.08) |
| Positive values indicate a better BCF or insulin sensitivity, negative values indicate a lower BCF or insulin sensitivity.  1: Crude model adjusted for sex, age and insulin sensitivity  2: model 1 + education, blood pressure expressed in MAP, lipid-modifying medication, antihypertensive medication, moderate-to-vigorous physical activity, high-density cholesterol, low-density cholesterol, triglycerides, intake of dietary fibre, *trans* fat, red meat and fruit intake, and mutual adjustment for the other beverage category  3: model 2 +total energy intake  4: fully adjusted model; model 3 + body mass index  * Matsuda index is not adjusted for insulin sensitivity | | | | |

| **Appendix Table 4:** Associations of artificially and sugar-sweetened beverage intake with β-cell rate sensitivity tertiles(odds ratios and 95% confidence intervals) | | | | |
| --- | --- | --- | --- | --- |
|  | **Tertile 1 versus 3 β-cell rate sensitivity** | | | |
|  |  | **Non consumers**  **(ref)** | **Moderate consumers** | **Daily consumers** |
|  | Model | OR | OR (95%CI) | OR (95%CI) |
| **Artificially sweetened soft drink** |  |  |  |  |
| N |  | *1187* | *887* | *166* |
|  | 1 | 1 | 1.35 (1.09, 1.69) | 1.54 (1.09, 1.69) |
|  | 2 | 1 | 1.29 (1.03, 1.61) | 1.24 (0.82, 1.87) |
|  | 3 | 1 | 1.29 (1.03, 1.61) | 1.24 (0.82, 1.87) |
|  | 4 | 1 | 1.29 (1.03, 1.62) | 1.26 (0.83, 1.91) |
| **Sugar-sweetened soft drink** |  |  |  |  |
| N |  | *825* | 1299 | *116* |
|  | 1 | 1 | 0.90 (0.72, 1.12) | 1.47 (0.91, 2.38) |
|  | 2 | 1 | 0.95 (0.75, 1.20) | 1.40 (0.85, 2.31) |
|  | 3 | 1 | 0.96 (0.76, 1.21) | 1.46 (0.87, 2.43) |
|  | 4 | 1 | 0.95 (0.75, 1.21) | 1.46 (0.87, 2.43) |
| **Juice** |  |  |  |  |
| N |  | *274* | *1851* | *115* |
|  | 1 | 1 | 0.69 (0.50, 0.95) | 0.59 (0.35, 1.02) |
|  | 2 | 1 | 0.82 (0.59, 1.140) | 0.71 (0.41, 1.25) |
|  | 3 | 1 | 0.82 (0.59, 1.15) | 0.73 (0.41, 1.28) |
|  | 4 | 1 | 0.82 (0.59, 1.15) | 0.72 (0.41, 1.27) |
| **Total sugar-sweetened beverage** |  |  |  |  |
| N |  | *145* | *1689* | *406* |
|  | 1 | 1 | 0.66 (0.43, 1.01) | 0.85 (0.53, 1.38) |
|  | 2 | 1 | 0.77 (0.49, 1.19) | 0.95 (0.585, 1.536) |
|  | 3 | 1 | 0.77 (0.50, 1.19) | 0.99 (0.60, 1.63) |
|  | 4 | 1 | 0.77 (0.50, 1.19) | 0.99 (0.60, 1.63) |
| Third tertile of rate sensitivity is the reference group (best rate sensitivity)  Values <1.00 indicate a better β-cell rate sensitivity, values >1.00 indicate a lower β-cell rate sensitivity.  1: Crude model adjusted for sex, age and insulin sensitivity  2: model 1 education, blood pressure expressed in MAP, lipid-modifying medication, antihypertensive medication, moderate-to-vigorous physical activity, high-density cholesterol, low-density cholesterol, triglycerides, intake of dietary fibre, *trans* fat, red meat and fruit intake, and mutual adjustment for the other beverage category  3: model 2 +total energy intake  4: fully adjusted model; model 3 + body mass index | | | | |
